# Anti-Parasitic Activity of Cherry Tomato Peel Powders

**DOI:** 10.3390/foods10020230

**Published:** 2021-01-23

**Authors:** Mendel Friedman, Christina C. Tam, Jong H. Kim, Sydney Escobar, Steven Gong, Max Liu, Xuan Yu Mao, Cindy Do, Irene Kuang, Kelvin Boateng, Janica Ha, Megan Tran, Srimanth Alluri, Tam Le, Ryan Leong, Luisa W. Cheng, Kirkwood M. Land

**Affiliations:** 1 Healthy Processed Foods Research Unit, Agricultural Research Service, United States Department of Agriculture, Albany, CA 94710, USA; 2 Foodborne Toxins Detection and Prevention Research Unit, Agricultural Research Service, United States Department of Agriculture, Albany, CA 94710, USA; christina.tam@usda.gov (C.C.T.); jongheon.kim@usda.gov (J.H.K.); luisa.cheng@usda.gov (L.W.C.); 3 Department of Biological Sciences, University of the Pacific, Stockton, CA 95211, USA; s_escobar1@u.pacific.edu (S.E.); s_gong3@u.pacific.edu (S.G.); m_liu11@u.pacific.edu (M.L.); s_mao@u.pacific.edu (X.Y.M.); c_do4@u.pacific.edu (C.D.); i_kuang@u.pacific.edu (I.K.); k_boateng@u.pacific.edu (K.B.); j_ha8@u.pacific.edu (J.H.); m_tran59@u.pacific.edu (M.T.); s_alluri@u.pacific.edu (S.A.); yentam7160@gmail.com (T.L.); r_leong2@u.pacific.edu (R.L.); kland@pacific.edu (K.M.L.)

**Keywords:** cherry tomatoes, cherry tomato peels, cherry tomato pomace, anti-trichomonad properties, composition, food and industrial uses, animal health, human health, research needs

## Abstract

Trichomoniasis in humans, caused by the protozoal parasite *Trichomonas vaginalis,* is the most common non-viral sexually transmitted disease, while *Tritrichomonas foetus* causes trichomonosis, an infection of the gastrointestinal tract and diarrhea in farm animals and domesticated cats. As part of an effort to determine the inhibitory effects of plant-based extracts and pure compounds, seven commercially available cherry tomato varieties were hand-peeled, freeze-dried, and pounded into powders. The anti-trichomonad inhibitory activities of these peel powders at 0.02% concentration determined using an in vitro cell assay varied widely from 0.0% to 66.7% against *T. vaginalis* G3 (human); from 0.9% to 66.8% for *T. foetus* C1 (feline); and from 0.0% to 81.3% for *T. foetus* D1 (bovine). The organic *Solanum lycopersicum* var. *cerasiforme* (D) peels were the most active against all three trichomonads, inhibiting 52.2% (G3), 66.8% (C1), and 81.3% (D1). Additional assays showed that none of the powders inhibited the growth of foodborne pathogenic bacteria, pathogenic fungi, or non-pathogenic lactobacilli. Tomato peel and pomace powders with high content of described biologically active compounds could serve as functional food and feed additives that might help overcome adverse effects of wide-ranging diseases and complement the treatment of parasites with the anti-trichomonad drug metronidazole.

## 1. Introduction

Tomatoes are a low-fat food source containing nutrients that include amino acids, unsaturated fatty acids, dietary fiber, pectin, vitamins, and minerals. As noted elsewhere [1], tomatoes also produce secondary metabolites including phenolic compounds, flavonoids, pigments (β-carotene, lycopene), phytoalexins, protease inhibitors, and glycoalkaloids. The metabolites help protect the plant against adverse effects of phytopathogenic fungi, bacteria, viruses, and insects, and have also been shown to have beneficial health effects in animals and humans. 

According to the Food and Agricultural Organization of the United Nations [2], the worldwide production of tomatoes is continually increasing, approximately doubling from the year 1997 to 2017. Asia is reported to produce 54% of the total, Europe 15.7%, the Americas 17.6%, and Africa 12%. Both whole tomatoes and processed products, including tomato juice, paste and sauces, and canned dried tomatoes, are widely consumed foods. The generation of tomato-based products is accompanied by the formation of tomato pomace, consisting of tomato peels and seeds. Lu et al. [3] reviewed worldwide efforts designed to facilitate the utilization of tomato pomace as an animal feed and as a human functional food that can be added to plant- and meat-based foods.

As part of a research effort designed to discover health-promoting properties of plant extracts and their biologically active compounds to inhibit the growth of pathogenic protozoa that cause human and animal diseases, we previously reported in several publications related to the present study that the tomato glycoalkaloid tomatine [4], theaflavin-rich black tea extracts [5], potato peels, and their glycoalkaloids α-chaconine and α-solanine [6], and structurally different anthraquinones [7] inhibited in cell assays the growth of *Trichomonas vaginalis* human strain G3 that causes the sexually transmitted disease trichomoniasis mostly in women, but also in some men, *Tritrichomonas foetus* bovine strain D1 that causes the sexually transmitted disease in farm animals (bulls, cows, and pigs) called trichomonosis, and *Tritrichomonas feline* strain C1 that causes intestinal diarrhea via the oral route in domestic animals (cats and dogs), reviewed in [8]. We suggested that these naturally occurring trichomonad formulations have the potential to complement or replace the widely used synthetic drug metronidazole that is reported to exhibit a number of adverse effects and the efficacy of which is reported to be diminishing against resistant trichomonad strains [9]. The present study extends the previous anti-trichomonad results by determining the inhibitory properties of seven peel powders prepared from commercially obtained cherry (grape) tomatoes, both grown organically and non-organically, to determine if there may be differential activities associated with cultivation methods. The reported content of biologically active compounds in cherry and standard (non-cherry) tomatoes are also briefly reviewed to facilitate insights into possible relationships between the nature and levels of bioactive tomato compounds and anti-trichomonad and other reported health benefits and to stimulate needed further research. 

## 2. Materials and Methods

### 2.1. Plant Material, Parasite, Bacterial, and Fungal Strain Source

*Trichomonas vaginalis* human strain G3 was from Patricia Johnson, University of California at Los Angeles, CA, *Tritrichomonas foetus* feline strain D1 was from Lynette Corbeil at the University of California at San Diego, La Jolla, CA, USA, and feline *Trichomonas foetus*-like organism (strain C1) was from Stanley Marks, University of California at Davis, School of Veterinary Medicine, Davis, CA, USA. The pathogenic and nonpathogenic bacteria and pathogenic fungi were obtained from the in-house United States Department of Agriculture (USDA) collection or from the American Type Culture Collection (ATCC, Manassas, VA, USA). The following seven varieties of fresh cherry tomatoes were obtained from local stores in California: *Solanum lycopersicum* ‘Campari’ non-organic, non-organic *Solanum lycopersicum* ‘Kumato’, non-organic *Solanum lycopersicum* var. *cerasiforme* (A), organic *Solanum lycopersicum* var. *cerasiforme* (B), organic *Solanum lycopersicum* var. *cerasiforme* (C), organic *Solanum lycopersicum* ‘Roma’, organic *Solanum lycopersicum* var. *cerasiforme* (D).

### 2.2. Preparation of Cherry Tomato Peel Powders

The cherry tomatoes were purchased at local stores in California. They were subjected to blanching before peeling as follows. After manual removal of the stems, they were washed with water using a brush. An X was then cut with a knife into the bottom of each tomato, which were then placed into a saucepan with boiling water for 60 s. The tomatoes were removed using a large spoon and placed into a container with ice water. The loose peels of the cooled, wrinkled blanched tomatoes were then removed manually. All wet peels were freeze-dried (lyophilized) and the resulting dry peels were then ground to fine powders using an electric coffee grinder (Kreps, Millville, NJ, USA). The final yields of the peel powders, about 10–13%, were determined from the original weights of the fruits and vegetables and those of the freeze-dried peel powders (Table 1). For example, the red powder from 20.5 g of organic red grape tomatoes weighed 2.7 g, corresponding to a yield of 12.6%.

### 2.3. Stock Solutions for Determination of Anti-Trichomonad Effects 

The cherry peel powders were dissolved in a 1:1 solution of dimethyl sulfoxide (DMSO): water to a concentration of 0.02%. This solution was used as the solvent because it facilitates solubilization of test substances and has been used in our previous studies. Solutions were prepared fresh and vortexed immediately before use.

### 2.4. Trichomonad Growth Inhibition Assays

Cultures of the G3 strain of *T. vaginalis* and C1 and D1 strains of *T. foetus* were grown and maintained in 11 mL of TYM Diamond medium of pH 6.2. Every 24 h, the cells from the C1, D1, and G3 strains were passed by inoculating 1000 μL of cells (approximately 1 × 10^6^ cells) into a new 15 mL conical tube containing 10 mL of TYM Diamond medium. Then, the cells were incubated for 24 h at 37 °C. Inhibitory screens were carried out as previously described [4,5,6]. These assays were incubated at 37 °C for 24 h before being counted using a hemocytometer. Stock solutions were then diluted in media and tested over a range of increasing concentrations. Percentage inhibitory activities were calculated relative to the DMSO:water control at the same concentration as the test substances. There was very little if any toxicity associated with this DMSO:water solvent vehicle control.

### 2.5. Bacterial and Fungal Screens

The cherry peel powders were evaluated for their antibacterial properties in disc diffusion growth assays at a concentration of 10% *w*/*v* in DMSO:water (1:1). The negative control was 1:1 DMSO:water. Positive controls for growth inhibition for the various bacteria were discs containing the following antibiotics: levofloxacin 5 µg, gentamicin 10 µg, and gentamicin 120 µg. Agar plates were incubated at 37 °C for 18–24 h and zones of inhibition in millimeters (mm) were measured. None of the compounds had antibacterial activity at the concentrations tested nor did the vehicle control DMSO:water. The antifungal activity was examined in *Aspergillus fumigatus* AF293, a causative agent for invasive aspergillosis, and *Candida albicans* ATCC10231. DMSO:water was used as a negative vehicle control and 5 mM octyl gallate was used for the positive control for the growth of both fungi. In both *A. fumigatus* and *C. albicans* tests, 5 µL of the test powders were spotted onto the lawn of fungi (in duplicate), which were grown on Potato Dextrose Agar (PDA) or Yeast Peptone Dextrose (YPD; Bacto yeast extract 1%, Bacto peptone 2%, glucose 2%) (Millipore Sigma, St. Louis, MO, USA) for *A. fumigatus* or *C. albicans*, respectively. Fungi were incubated at 35 °C and the formation of zones of inhibition in millimeters (mm) were monitored at 24 and 48 h. None of the cherry tomatoes showed antifungal activity against the two pathogenic fungi nor did the vehicle control DMSO:water have any effect.

### 2.6. Statistical Analysis

All parasite screening trials were performed a minimum of three times on three separate days to a standard error of ≤0.10. Statistical significance was determined using the Student’s *t*-test to generate *p* values in the Prism 6 software (GraphPad, San Diego, CA, USA). *p* values < 0.05 were considered statistically significant.

## 3. Results

### 3.1. Weight Changes during Transformations of Cherry Tomatoes to Dry Peels

Table 1 shows the observed changes in weights that have occurred during the preparation of seven cherry tomato powders from fresh cherry tomatoes. The data show that the weights of the wet peels following hand-peeling ranged from 4.6% (Organic *Solanum lycopersicum* var. *cerasiforme* C) to 7.9% or a 1.72-fold variation from the lowest to the highest value. The last two columns show the weight changes resulting from freeze-drying the wet to dry peels. The percentage change ranged from 4.7% (Organic *Solanum lycopersicum* var. *cerasiforme* D) to 13.6% (Organic *Solanum lycopersicum* var. *cerasiforme* B) or a 2.89-fold variation from lowest to highest value. The results show no apparent major differences in weight changes between the dry peel powders derived from the three non-organic and four-organic cherry tomatoes.

### 3.2. Growth Inhibition of Cherry Tomato Peel Powders against Trichomonad Parasites

**Figure 1:**
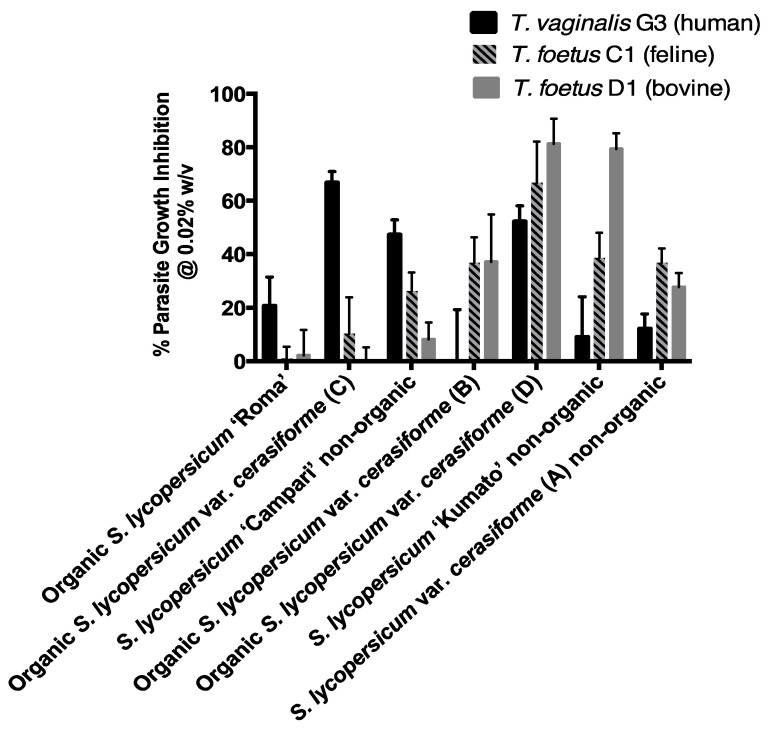
Cherry tomato powders and peels inhibit the growth of trichomonad parasites. The graph shows the percentage growth inhibition for *T. vaginalis* G3, *T. foetus* C1, and *T. foetus* D1 at the final concentration of 0.02% *w*/*v* for each powder and peel. Percentage growth inhibition was calculated relative to the DMSO:water vehicle control at the same concentration as the test substance in each experiment. There was very little if any toxicity associated with the vehicle control. Differential inhibition of parasites is observed.

Figure 1 and Table 2 show that at a concentration of 0.02% *w*/*v*, six of the seven evaluated peel powders inhibited the growth of *T. vaginalis* G3 (human). There was very little if any toxicity associated with the vehicle control DMSO:water at the same concentration as the test powders in each experiment. In terms of percent inhibition under the test conditions, the anti-trichomonad effects ranged from 9.0% (*Solanum lycopersicum* ‘Kumato’ non-organic) to 66.7% (Organic *Solanum lycopersicum* var. *cerasiforme* C) or a 7.4-fold variation from the lowest to highest value. The *Solanum lycopersicum* ‘Campari’ non-organic and Organic *Solanum lycopersicum* var. *cerasiforme* (D) varieties also inhibited the G3 strain at high levels of 47.2% and 52.2%, respectively. However, the Organic *Solanum lycopersicum* var. *cerasiforme* (B) peels were inactive. A similar analysis of the data for *T. foetus* C1 (feline) in Table 2 also shows a broad range of activities from the highest value of 66.8% (Organic *Solanum lycopersicum* var. *cerasiforme* D) to the lowest value of 0.9% (Organic *Solanum lycopersicum* ‘Roma’) or a 74.2-fold variation. The inhibitory values for the six other peel powders were intermediate, ranging from 10.2% (Organic *Solanum lycopersicum* var. *cerasiforme* C) to 38.7% (*Solanum lycopersicum* ‘Kumato’ non-organic)

The trends for *T. foetus* D1 (bovine) in the last column of Table 2 show that the inhibitory activities of the six peel powders ranged from 2.2% (organic *Solanum lycopersicum* ‘Roma’) to 81.3% (organic *Solanum lycopersicum* var. *cerasiforme* D) or a 40.1-fold variation. The *Solanum lycopersicum* ‘Kumato’ non-organic peels were also highly active, inhibiting 79.4% under the test conditions. The inhibitory activities of five other peels ranged from 8.2% (*Solanum lycopersicum* ‘Campari’ non-organic) to 37.2% (organic *Solanum lycopersicum* var. *cerasiforme* B). The organic *Solanum lycopersicum* var. *cerasiforme* (C) peels were inactive. The results show large variations among the inhibitory activities of the cherry peel powders, both within each trichomonad strain as well as between the three strains with one exception. The activity of the organic *Solanum lycopersicum* var. *cerasiforme* (D) peel powder was high against all three trichomonad strains, ranging from 52.2% for the human G3, to 66.8% for the feline C1, and 81.3% for the bovine D1 strain. Using the Student’s *t*-test for these percent inhibitory values for the Organic *Solanum lycopersicum* var. *cerasiforme* (D) peel powder in all three trichomonad strains, we found that there was a statistical significance between the *T. vaginalis* G3 (human) vs. the *T. foetus* D1 (bovine strain) (Table 2, *p* < 0.05) but not for the other combinations (Table 2, *p* > 0.05, *T. foetus* C1 feline vs. *T. foetus* D1 bovine or *T. vaginalis* G3 human vs. *T. foetus* C1 feline). This indicates that the *T. foetus* D1 (bovine) strain may be more susceptible to the inhibitory activities of the *Solanum lycopersicum* var. *cerasiforme* (D) peel powder in comparison to the other two trichomonad strains indicating strain susceptibility. The same statistical analysis was performed for the rest of the peel powders between all the three trichomonad strains and they are reflected in Table 2 with * and ^b^ superscripts to indicate statistical significance *p* < 0.05.

### 3.3. Screening of Antibacterial and Antifungal Activities of the Cherry Tomato Powders

We also determined possible inhibitory activities of the cherry tomato peel powders against four pathogenic bacteria as well as against three nonpathogenic (commensal) lactobacilli in disc diffusion assays. The results in Table 3 show that, compared to three standard antibiotics used as positive controls, none of the peel powders was active against the four pathogenic bacteria in this assay. Table 4 shows a lack of inhibitory activity for the tomato peels against the lactobacilli and *E. coli* as compared to the inactivation observed with the three antibiotics. Similar negative results were observed against two fungal pathogens as compared to the activity of the positive control octyl gallate (Table 5). Additionally as shown in all the tables, the DMSO:water solvent vehicle control had no toxicity effects on the strains tested as indicated with the 0 mm zones of inhibition.

## 4. Discussion

**Figure 2:**
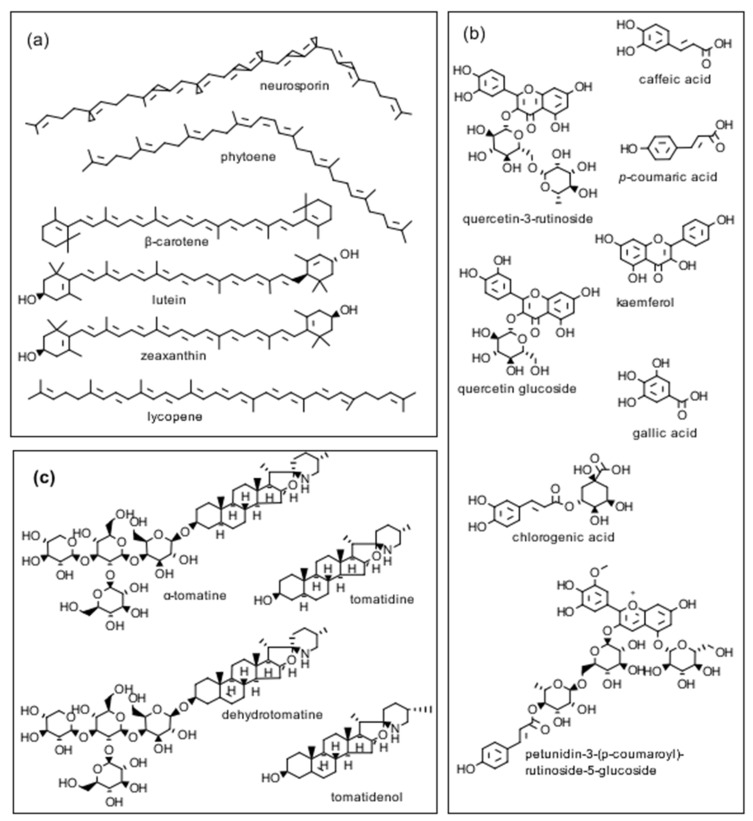
Structures of three classes of biologically active tomato compounds: (**a**) pigments, (**b**) phenolics, and (**c**) tomato glycoalkaloids discussed in the text.

As mentioned earlier, tomatoes are a low-fat food source, producing both nutrients as well as biologically active compounds that include glycoalkaloids, antioxidative phenolic compounds, flavonoids, carotenes, and lycopene, all of which have been reported to have health-promoting properties (Figure 2). In our study with the seven commercially available cherry tomato varieties (three non-organic, four organic), we found differential growth inhibitory activities amongst the different varieties. The organic *Solanum lycopersicum* var. *cerasiforme* (D) powder was potent against the growth of all three trichomonad strains with the best growth inhibition targeting *T. foetus* D1 (Bovine). Interestingly, only two other powders showed potency >50% growth inhibition against any of the three trichomonad strain: (a) organic *Solanum lycopersicum* var. *cerasiforme* (C) against *T. vaginalis* G3 (human) and (b) *Solanum lycopersicum* ‘Kumato’ non-organic against *T. foetus* D1 (Bovine). Our study could not determine any significant correlation relating to inhibitory activities against these three protozoan parasites and the method of cultivation (i.e., organic vs. non-organic). However, this study was complicated as these seven commercially available cherry tomato varieties were not the same ‘cultivars’, and genetic differences may determine the production or lack of production of important biologically active compounds that could affect our study. It is also worth noting that the drying technology used in an industrial exploitation of the results could lead to different levels of bioactivity because small-scale freeze-drying of the wet peels used in the present study is probably too expensive to be used to dry tomato peels on a large scale. In the sections below, we will discuss some important properties of tomatoes that can impact their use as food additives or functional foods.

### 4.1. Content of Biologically Active Compounds in Cherry Tomatoes, Peels, and Pomaces

#### 4.1.1. General Properties

The properties of the biologically active compounds include antibiotic, anticarcinogenic, cardioprotective, and antilipidemic effects [10]. Both whole tomatoes and processed products that include tomato juice, paste, and sauces are widely consumed around the world. The generation of these products is accompanied by the formation of tomato pomace, considered to be a waste product, which consists of tomato peels and seeds. To facilitate the assessment of the possible relationship between the levels of biologically active compounds in tomato varieties to their potential health benefits, we present highlights from the literature on the composition of selected, predominantly cherry, tomato varieties, and genetically modified tomatoes, as well on the potential food and industrial uses of cherry tomato peels and pomace. The content of these compounds in different cherry and other tomato plants briefly outlined below might help our incomplete understanding of possible relationships between composition and bioactivity.

#### 4.1.2. Analytical Aspects

Here, we briefly summarize reported studies on the content of bioactive compounds in cherry tomatoes.

A comparison of standard and rapid extraction and chromatographic separation methods for tomato carotenoids of cherry and processing tomatoes by Dzakovich et al. [11] suggests that the rapid extraction and high-performance liquid chromatography (HPLC)-diode array detector (HPLC-DAD) methods could enhance throughput for some uses compared with standard protocols. Kozukue et al. [12] reported on the analysis of the tetrasaccharide glycoalkaloids α-tomatine and dehydrotomatine as well the corresponding aglycones tomatidine and tomatidenol lacking the carbohydrate side chain, as illustrated in Figure 2 of vegetative parts (calyxes, flowers, green and red tomato fruit, leaves, roots and stems) of greenhouse-grown cherry (mini) and two standard tomatoes using HPLC and mass spectrometry. The analytical method for tomatidine is of interest because it can be used in metabolism and pharmacology studies of reported beneficial biological properties, including anti-trichomonad [4], antiviral [13], anti-inflammatory [14], and anti-cancer effects [15]. Friedman et al. [16] reported on the analysis of peel powders from six commercial cherry tomato varieties for free amino acid, phenolic, flavonoid, β-carotene, lycopene, and glycoalkaloid content using HPLC/MS (mass spectrometry). In a related analytical study, we reported that the sums of the two glycoalkaloids dehydrotomatine and α-tomatine in tomato stems, green tomato peel, and tomato leaves harvested from a tomato plant (in mg/g dry weight, DW) are 5.14, 12.4, and 16.4, respectively [17]. These results suggest that the leaf powder might show useful biological activity.

Choi et al. [18] determined the content of water, free amino acids, amino acid metabolites, crude protein, the carotene pigments β-carotene and lycopene, and 9 characterized and 2 incompletely characterized individual phenolic (flavonoid) compounds of 12 greenhouse-grown cherry tomato varieties of various colors (green, yellow, orange, red, and black) using HPLC and LC/MS methods. The study also found that the phenolic content of the cherry tomatoes per unit weight is 3–4 times greater than reported values for large-sized tomatoes and that lycopene showed strong activity against cervical carcinoma and lung cancer cells. Bagley et al. [19] carried out a metabolomic analysis of commercial cherry tomatoes using infrared matrix-assisted laser desorption electrospray ionization (IR-M ALDESI) mass spectrometry imaging analysis. The results revealed the presence of 1626 unique compounds that included new cyanogenic glycosides, glucosinolates, and lignans. A study by Coyago-Cruz et al. [20] of the commercial quality parameters of five cherry and six non cherry tomato varieties showed that, for the cherry tomatoes, the total carotenoid content (consisting of phytoene, phytofluene, lutein, and lycopene, a β-carotene) ranged (in mg/100 g DW) from 2.5 to 102.0. The corresponding range for six phenolic compounds (p-hydroxybenzoic, caffeic, chlorogenic, p-coumaric, and gallic acids and quercetin was from 150.2 to 307.7 mg/100 g DW, and for three sugars (fructose, glucose, sucrose from 308.4 to 524.1. The authors suggest that the results provide useful information on the biosynthesis and bioavailability of tomato carotenoids.

Naviglio et al. [21,22] developed an innovative low-cost high-pressure water extraction process for the *trans*-lycopene isomer from industrial tomato byproducts with 98% (*w*/*w*) purity and up to 14% (*w*/*w*) recovery. Silva et al. [23] used an innovative hydrophobic eutectic solvent to extract lycopene from tomato processing byproducts, suggesting that the method can also contribute to the development of greener extraction processes that can replace the use of organic solvents. Horuz et al. [24] used an electrospinning method to encapsulate carotenoids extracted from tomato peel into zein nanofibers, resulting in their improved storage and thermal stability and in an 11-fold increase in antioxidant activity.

The cited results demonstrate both wide-ranging differences as well as similarities in the content of nutritional and bioactive compounds in cherry tomatoes, and suggest that such knowledge can benefit consumers, who could select cherry tomatoes with the highest health-promoting properties. The methods described and associated results could also aid in the targeted development of functional foods containing cherry tomato peels with health-improving properties.

#### 4.1.3. Preharvest Changes of Composition during Growth of Tomato Plants

Coyago-Cruz et al. [25] assessed the relationship between the effect of regulated deficit irrigation, cluster, developmental stages and two growth seasons on the commercial and functional quality (carotenoids and phenolic levels) of cherry tomatoes. The highest carotenoids and phenolic levels were found in the higher clusters higher up in the plant and carotenoids in ripe fruit, suggesting that irrigation of such varieties could be reduced by 80% without affecting considerably the overall fruit quality. Choi et al. [26] analyzed 11 Korean tomato varieties grown under the same greenhouse conditions and 13 processed commercial tomato products for the content of free amino acids, amino acid metabolites, protein, individual phenolics, total phenolics, and for their antioxidative activity and cancer cell-inhibiting effects. The results show a broad range of bioactive compounds across tomato varieties and products. The results of another study by Choi et al. [27] on changes in the composition of tomatoes during eleven stages of growth (S1–S11) show that the total content (in mg/100 g of fresh weight, FW) of the free amino acids ranged from 41 to 85 in the green tomato extracts S1–S7 and then increased to 251 (S9) in the red, followed by a decrease to 124 in S11 red; the total initial concentration of up to 12 phenolic compounds of 2000 µg/100 g of FW varied throughout the ripening process, with the quantity decreasing and the number of compounds increasing in the red tomato; chlorophyll a and b content of tomatoes harvested during S1 was 5.73 mg/100 g FW pericarp and then decreased continuously to 1.14 mg/100 g for S11; the concentration (in mg/100 g of FW) of lycopene in the S8 red of 0.32 increased to 1.27 in S11; and tomatoes harvested during S1 contained 48.2 mg of dehydrotomatine/100 g of FW, and this value continually decreased to 1.5 in S7, with no detectable levels in S8–S11. The corresponding α-tomatine content decreased from S1 (361) to S8 (13.8).

It seems that the harvesting stage significantly affects the composition of tomatoes, suggesting that it would benefit human health to consume both green and red tomatoes with complementary bioactive compounds. The described results may also make it possible to better relate the structures of the active compounds to their health-promoting function, individually, in combination, and in food, and allow the consumer to select food with the optimal content of nontoxic beneficial compounds.

#### 4.1.4. Formation of New Bioactive Tomato Compounds via Plant Genetics

An assessment by da Silva Souza et al. [28] of the composition of two allele-introgressed tomato lines show that a purple tomato peel accumulated high amounts of the anthocyanin petunidin 3-(p-coumaroyl)-rutinoside-5-glucoside (Figure 2) and the flavonoids rutin and kaempferol, while the orange tomato accumulated high amount of β-carotene, and had a reduced lycopene level. The purple tomatoes had the highest phenolic content. Campestrini et al. [29] reported that the lutein, lycopene, and β-carotene content of a new purple tomato variety was 6, 1.5, and 2.5 times greater than that of a commercial cherry tomato variety. The authors suggest that both varieties are good sources of dietary carotenoids with health-promoting antioxidant and cancer cell inhibiting properties. 

The carotenoid zeaxanthin in combination with lutein (Figure 2), are reported to delay progression of age-related macular degeneration of the retina in the human eye by filtering damaging blue light. Karniel et al. [30] used two genetic approaches to create a new tomato variety named ‘Xantoato’ containing 39 µg/g of FW (577 µg/g DW) of zeaxanthin that constitutes 50% of total carotenoids in the fruit,

#### 4.1.5. Effect of Heat, Methyl Jasmonate, and Antimicrobial Coatings on Composition

An investigation by D’Evoli et al. [31] on the effect of heat treatment on the carotenoid content of cherry tomatoes showed that the lycopene content in canned tomatoes was two-fold higher than in raw tomatoes (11.60 mg/100 g vs. 5.12 mg/100 g). The lutein and β-carotene were, respectively, 0.15 mg/100 g and 0.75 mg/100 g in canned vs. 0.11 mg/100 g and 1.00 mg/100 g in raw tomatoes. Obadina et al. [32] investigated the quality changes of commercial cherry and plum tomatoes dried at different temperatures (60, 65, and 70 °C), then milled into powders and stored for 8 weeks. They found that the ascorbic acid content and lycopene content of the tomato powders were significantly different, with values that ranged from 5.10 to 7.70 mg/100 g and 211.53 to 246.02 mg/kg, respectively. Liu et al. [33] found that postharvest treatment of cherry tomatoes with the plant hormone methyl jasmonate stored for 11 days at room temperature significantly increased the content of ascorbic acid and carotenoids, especially lycopene, as well as of volatile organic compounds in the tomato fruits, but had no effect on firmness, sugars, and titratable acidity. These observations suggest that the treatment can enhance the health benefits of the cherry tomatoes. The naturally occurring compound methyl jasmonate has also been reported to induce cell death in *T. vaginalis* [34,35]. Guo et al. [36] reported that carboxymethylcellulose-based films with added cinnamaldehyde and zinc oxide with antifungal activity against *Aspergillus niger* were significantly effective at inhibiting the weight loss and firmness of cherry tomatoes and in decreasing their total acidity after storage.

These observations are consistent with our reported antibiotic activity of cinnamaldehyde in fruit and vegetable edible films against leafy greens contaminated with pathogenic *E. coli* bacteria [37]. Will the edible films also protect cherry tomatoes against foodborne pathogens?

### 4.2. Food and Industrial Uses of Tomato Pomace

Nour et al. [38] reported that supplementation of wheat flour with 6% (*w*/*w*) of dry tomato waste powder resulted, after baking, in the production of bread with good sensory properties and acceptability. The authors suggest that the tomato waste containing ascorbic acid, β-carotene, lycopene, phenolic compounds, and minerals can be used as functional ingredient for the formulation of antioxidant-rich functional foods. A related study by Bajerska et al. [39] found that a high-fat diet supplemented with rye bread enriched with tomato pomace fed to rats seems to lower the atherogenic index of plasma as well as the total lipid content of the liver, suggesting that tomato pomace affects the energy balance via fecal loss of lipids. Crawford et al. [40] found that baked flatbreads prepared with quinoa flour supplemented with tomato peel powders have the potential to serve as a high nutritional, gluten-free, low-acrylamide health-promoting functional food. Raviv et al. [41] discovered that cherry tomatoes added to compost reduced the incidence of crown and root-rot disease caused by *Meloidogyne javanica* as well as the population size of the causal pathogen *Fusarium oxysporum* radicis-lycopersici. Tommonaro et al. [42] reported that industrial processing of cherry tomatoes resulted in decrease of antioxidant activity compared with that of the fresh fruit, and that a higher polyphenolic content was present in tomato juice containing peels and seeds in comparison to those without, suggesting that tomato juice prepared from cherry tomatoes might serve a useful and health-benefitting source of antioxidants in the diet. Stajčić et al. [43] determined the carotenoid content, antioxidant and growth-inhibitory activities against cancer cells of tomato waste extracts consisting of tomato peels and seeds from five different tomato genotypes. The carotenoid content was strongly correlated with the antioxidant and anti-proliferation activity. Qiu and Chin [44] found that covering low-fat pork sausages with an edible film prepared using sodium alginate and cherry tomato powder resulted in reduced lipid oxidation and microbial growth as well as in extended shelf like of the sausages. Finally, Sabio et al. [45] determined the effect of multiple variables including temperature, residence time, and biomass/water ratio on the hydrothermal carbonization of tomato peel waste into a solid hydrocarbon fuel. 

The described results show that the levels of bioactive components of cherry tomatoes, tomato peels, and tomato pomaces described here vary widely and are influenced by multiple factors. Both the nature and content of biologically active tomato compounds depend on tomato variety as well as on both environmental (soil) preharvest and postharvest storage and processing of the harvested tomatoes. The cited studies described here and associated results offer insight into the multiple factors that affect the composition and biological properties of the tomato compounds shown in Figure 2, and could aid in the development of functional foods with health-improving properties, as well as in the transformation of tomato peels and pomaces to value-added industrial products.

### 4.3. Nutritional and Health Benefits of Tomato Peels and Pomaces

Vats et al. [46] suggested that tomato wastes consisting of peel and seed extracts are rich in proteins and oil that can help alleviate global malnutrition. Espinosa-Juárez et al. [47] showed that feeding high-sucrose (obese) male rats extracts of tomato pomace had a beneficial effect on lower urinary tract symptoms by enhancing prostate function. Mansoori et al. [48] found that feeding hens dried tomato pomace and alfalfa meal was effective in inducing moult, while reducing stress of severe starvation and retaining egg quality. Romano et al. [49] determined the effect of feeding sheep tomato byproducts on the composition of the resulting milk fat distribution compared to those fed a standard diet. The content of conjugated linoleic acid content (CLA) of 19.8% and of the polyunsaturated (PUFA) of 6.43% were higher than observed with the standard diet. A related study by Abbeddou et al. [50] discovered that feeding tomato pomace containing diets to lactating ewes over 7 weeks resulted in decreased saturated and polyunsaturated fatty acid and in increased content of monounsaturated including 18:1 trans fatty acids in the milk [51,52].The aggregation platelets in blood vessels play an important function in hemostasis and thrombosis associated with cardiovascular disease [53] as well as in innate immunity and in the regulation of growth of blood vessel tumors [54]. A clinical study by Palomo et al. [55] found that daily consumption of 1 g of aqueous extract of tomato pomace for 5 days inhibited platelet aggregation, suggesting its value to improving heart health.

## 5. Conclusions

This investigation has shown that some of the powders prepared from cherry tomato peels have anti-trichomonad, but not antibacterial or antifungal properties in vitro. Our results suggest that the organic *Solanum lycopersicum* var. *cerasiforme* (D) peel powder was effective in inhibiting the growth of all three trichomonad strains with ≥ 50%. We found that there was a statistical significance between the *T. vaginalis* G3 (human) vs. the *T. foetus* D1 (bovine strain) (Table 2, *p* < 0.05) but not for the other combinations (Table 2, *p* > 0.05, *T. foetus* C1 feline vs. *T. foetus* D1 bovine or *T. vaginalis* G3 human vs. *T. foetus* C1 feline). This indicates that the *T. foetus* D1 (bovine) strain may be more susceptible to the inhibitory activities of the *S. lycopersicum* var. *cerasiforme* (D) peel powder in comparison to the other two trichomonad strains indicating strain susceptibility. These results in conjunction with previously published studies suggest their potential value to ameliorate the severity of trichomoniasis in infected humans and trichomoniasis in infected farm and domestic animals. The results also suggest the need for the following further studies that might help broaden the value of cherry tomato fruit, peels, and pomaces for ameliorating human and animal diseases. These include (a) clinical trials on the effectiveness of cherry tomato peels with the highest content of biologically active compounds; (b) defining the effectiveness of the peel powders against metronidazole-resistant trichomonads; (c) determining whether animal feed or human food, such as breads and flatbreads [40], and supplemented with the cherry peel powders, might act as functional feeds and foods to help protect animals and humans against trichomoniasis; (d) developing novel methods to protect cherry tomatoes on growing plants against deterioration (spoilage) induced by phytopathogenic fungi, including *Alternaria alternata* [56] and aflatoxin-producing *Aspergillus flavus* [57,58,59]; and (e) determining anti-trichomonad potencies of peels from new tomato varieties that contain bioactive compounds developed via plant genetics [28,30]. 

We agree with the need for the following suggested further studies by a journal reviewer. “As stated by the authors, in virtue of the results here produced, one of the possible applications of peel powders would be its supplementation in foods, to ultimately protect animals and humans against trichomoniasis. Before in vivo trials, it is mandatory for a food chemist to prove, that salivary, gastric, and intestinal digestion, as well as metabolic processing, does not alter the anti-microbial activity of tomato peels. This experiment can be easily performed in an in vitro setting, where after each stage of simulated in vitro digestion, the processed extract is tested for anti-microbial activity. The authors should try also subjecting peel powders to metabolic processing, i.e., treating the extract with S9 microsomal fraction from rat liver. All the reagents necessary (salivary enzymes, intestinal enzymes, bile acids, ready-to-use S9 fraction) for this experiment are easy to obtain and commercially available. Adding the results of these experiments to the manuscript would augment its impact and give more insights concerning the activity of the extract. In vitro simulated digestion, for example, removes sugars from secondary metabolites. An eventual loss of anti-microbial activity would suggest the active molecules being glycosylated compounds. Paradoxically, after in vitro digestion, the peel extract could even acquire that antimicrobic or anti-fungi activity the undigested tomato peels was missing”. We are challenged to respond to the mentioned research needs.

## Figures and Tables

**Table 1:** Weights of fresh cherry tomatoes, wet peels, and peel powders.

Fresh Tomatoes (A) (g)	Wet Peels (B) (g)	Peel Powders (C) (g)	(C/B) × 100 (D) %
*Solanum lycopersicum* ‘Campari’ non-organic, 448	27.4 (6.8) ^a^	2.7	9.9 ^b^
*Solanum lycopersicum* ‘Kumato’ non-organic, 896	64.2 (7.8)	7.9	12.3
*Solanum lycopersicum* var. *cerasiforme* (A) non-organic, 896	70.6 (7.9)	7.3	10.3
Organic *Solanum lycopersicum* var. *cerasiforme* (B), 896	60.8 (6.8)	8.3	13.6
Organic *Solanum lycopersicum* var. *cerasiforme* (C), 448	20.5 (4.6)	2.6	12.7
Organic *Solanum lycopersicum* ‘Roma’, 896	53.8 (6.0)	5.5	10.2
Organic *Solanum lycopersicum* var. *cerasiforme* (D), 1344	62.7 (4.7)	4.1	4.7

^a^ Values in parenthesis are percent of wet peels recovered from peeling of fresh cherry tomatoes = (B/A) × 100. ^b^ Percent of peel powders recovered after freeze-drying wet peels.

**Table 2:** Activity of cherry tomato peel powders against three pathogenic trichomonads. The table shows the percentage growth inhibition ± standard deviations, *n* = 3, for *T. vaginalis* G3, *T. foetus* C1, and *T. foetus* D1 at the final concentration of 0.02% *w*/*v* for each peel powder. Percentage growth inhibition was calculated relative to the DMSO:water vehicle control at the same concentration as the test substance in each experiment. There was very little if any toxicity associated with the vehicle control. Student’s *t*-test were performed for each powder to determine the statistically significance of the percent growth inhibition values for each of the three trichomonad strains to each other. *p* < 0.05 were considered statistically significant.* *p* < 0.05 for *T. foetus* C1 (feline) and *T. foetus* D1 (bovine) compared against *T. vaginalis* G3 (human); ^b^ *p* < 0.05 comparison between *T. foetus* D1 (bovine) and *T. foetus* C1 (feline).

Cherry Tomato Names	*T. vaginalis* G3 (Human)	*T. foetus* C1 (Feline)	*T. foetus* D1 (Bovine)
*Solanum lycopersicum* ‘Campari’ non-organic	47.2 ± 5.7	26.3 ± 6.8 *^,b^	8.2 ± 6.3 *
*Solanum lycopersicum* ‘Kumato’ non-organic	9.0 ± 15	38.7 ± 9.3 *^,b^	79.4 ± 5.9 *
*Solanum lycopersicum* var. *cerasiforme* (A) non-organic	12.2 ± 5.6	36.9 ± 5.3 *	27.7 ± 5.3 *
Organic *Solanum lycopersicum* var. *cerasiforme* (B)	0.0 ± 19	36.9 ± 9.5 *	37.2 ± 18
Organic *Solanum lycopersicum* var. *cerasiforme* (C)	66.7 ± 4.2	10.2 ± 14 *	0.0 ± 5.3 *
Organic *Solanum lycopersicum* ‘Roma’	21.0 ± 11	0.9 ± 4.5 *	2.2 ± 9.6
Organic *Solanum lycopersicum* var. *cerasiforme* (D)	52.2 ± 5.9	66.8 ± 15	81.3 ± 9.4 *

**Table 3:** The zones of inhibition in mm (growth inhibition) from a disc diffusion assay targeted against bacterial pathogens using DMSO:water vehicle control, antibiotic controls, and test compounds at 10% *w*/*v* in DMSO:water.

	*Salmonella* *enterica*	*Listeria* *monocytogenes*	*Staphylococcus aureus*	*Bacillus* *cereus*
DMSO:water	0	0	0	0
Levofloxacin 5 µg	21	19	18	22
Gentamicin 10 µg	18	17	13	15
Gentamicin 120 µg	20	27	25	21
*Solanum lycopersicum* ‘Campari’ non-organic	0	0	0	0
*Solanum lycopersicum* ‘Kumato’ non-organic	0	0	0	0
*Solanum lycopersicum* var. *cerasiforme* (A) non-organic	0	0	0	0
Organic *Solanum lycopersicum* var. *cerasiforme* (B)	0	0	0	0
Organic *Solanum lycopersicum* var. *cerasiforme* (C)	0	0	0	0
Organic *Solanum lycopersicum* ‘Roma’	0	0	0	0
Organic *Solanum lycopersicum* var. *cerasiforme* (D)	0	0	0	0

**Table 4:** The zones of inhibition in mm (growth inhibition) from a disc diffusion assay targeted against bacterial commensals using DMSO:water vehicle control, antibiotic controls, and test compounds at 10% *w*/*v* in DMSO:water.

	*Escherichia coli* K12	*Lactobacillus acidophilus*	*Lactobacillus rhamnosus* GG	*Lactobacillus reuteri*
DMSO:water	0	0	0	0
Levofloxacin 5 µg	20	0	16	7
Gentamicin 10 µg	15	11	7	11
Gentamicin 120 µg	22	20	13	22
*Solanum lycopersicum* ‘Campari’ non-organic	0	0	0	0
*Solanum lycopersicum* ‘Kumato’ non-organic	0	0	0	0
*Solanum lycopersicum* var. *cerasiforme* (A) non-organic	0	0	0	0
Organic *Solanum lycopersicum* var. *cerasiforme* (B)	0	0	0	0
Organic *Solanum lycopersicum* var. *cerasiforme* (C)	0	0	0	0
Organic *Solanum lycopersicum* ‘Roma’	0	0	0	0
Organic *Solanum lycopersicum* var. *cerasiforme* (D)	0	0	0	0

**Table 5:** Activity of cherry tomato powders and peels against pathogenic fungi. The data indicate the calculated zone of inhibition (mm) for each powder and peel at the designated concentration 5 μL of a 10% *w*/*v* in DMSO:water with the positive control (octyl gallate) at 5 mM.

	*A. fumigatus* AF293	*C. albicans* ATCC10231
*Solanum lycopersicum* ‘Campari’ non-organic	0.0	0.0
*Solanum lycopersicum* ‘Kumato’ non-organic	0.0	0.0
*Solanum lycopersicum* var. *cerasiforme* (A) non-organic	0.0	0.0
Organic *Solanum lycopersicum* var. *cerasiforme* (B)	0.0	0.0
Organic *Solanum lycopersicum* var. *cerasiforme* (C)	0.0	0.0
Organic *Solanum lycopersicum* ‘Roma’	0.0	0.0
Organic *Solanum lycopersicum* var. *cerasiforme* (D)	0.0	0.0
Octyl gallate	6.7	8.0
DMSO:water	0.0	0.0

## Data Availability

The data generated by this study is available in this paper.
